# Interpretable deep learning for improving cancer patient survival based on personal transcriptomes

**DOI:** 10.1038/s41598-023-38429-7

**Published:** 2023-07-13

**Authors:** Bo Sun, Liang Chen

**Affiliations:** Department of Quantitative and Computational Biology, University of Southern California, 1050 Childs Way, Los Angeles, CA 90089 USA

**Keywords:** Machine learning, Gene ontology, Data mining, Computational models, Predictive medicine

## Abstract

Precision medicine chooses the optimal drug for a patient by considering individual differences. With the tremendous amount of data accumulated for cancers, we develop an interpretable neural network to predict cancer patient survival based on drug prescriptions and personal transcriptomes (CancerIDP). The deep learning model achieves 96% classification accuracy in distinguishing short-lived from long-lived patients. The Pearson correlation between predicted and actual months-to-death values is as high as 0.937. About 27.4% of patients may survive longer with an alternative medicine chosen by our deep learning model. The median survival time of all patients can increase by 3.9 months. Our interpretable neural network model reveals the most discriminating pathways in the decision-making process, which will further facilitate mechanistic studies of drug development for cancers.

## Introduction

Deep learning techniques take many forms and have shown great promise in various fields^1–5^. There has been significant interest in applying deep learning to advance biological or biomedical sciences. For example, AlphaFold^6^ computationally predicts the protein structure at an unprecedented accuracy with an average error of approximately 1.6 angstroms. DCell^7^ predicts the impact of genetic mutations on cellular growth response. DrugCell^8^ embeds chemical structures of drugs into neural networks to predict the drug response of human cancer cell lines based on DNA point mutations. Besides their applications to cancer prognostics and therapeutics^9,10^, deep neural networks (DNN) have also been applied to cancer survival analysis^11,12^, focusing on integrating multimodal data^13^ and interpretability^14,15^. However, no deep-learning method has yet considered drug treatment information in survival prediction. By incorporating the relationship between drug treatment, transcriptome, and survival, we can advance the goal of delivering personalized medicine and improving cancer prognosis.

Patients diagnosed with the same cancer and driver mutations frequently show distinct clinical features and rarely have identical responses to treatments. Genetic, transcriptomic, and other clinicopathological parameters may affect patients' survival. For some tumor types, the most significant contribution was reported from the transcriptome^16^. How genotypes and phenotypes are intertwined in cancer clinicopathology remains unclear, but the effect of genetic and cytogenetic alterations ought to be reflected in gene expression. At the same time, environmental impacts are also reflected in transcriptomes^17,18^. With deep learning techniques, global gene expression profiling appears to be the most powerful predictor of clinical outcomes. Unlike DrugCell predicting relative cell growth from point mutations, we moved beyond the cell lines and aimed to predict the clinical survival time directly based on patients’ gene expression profiles and the chemical structures of their administered drugs. Our deep-learning model enables the optimal drug choice based on individual transcriptome data.

In this work, we performed interpretable deep learning on cancer data from The Cancer Genome Atlas (TCGA)^19^ involving 33 primary tumor types. Specifically, we obtained the gene expression of primary tumor patients from TCGA and their clinical records logged in the Genomic Data Commons (GDC) database, such as vital status, survival time, and prescribed clinical drugs. We developed a DNN and named it CancerIDP (Cancer Interpretable neural network based on Drug prescriptions and Personal transcriptomes). The system was trained to embed drug chemical structures and gene expression profiling in the feature space and then to predict the survival time based on those embeddings. The network architecture of CancerIDP is based on the Gene Ontology (GO) structure in human cells, similar to DrugCell.

Our model is highly predictive regarding cancer patient survival: the prediction is accurate for the hold-out testing data. We report a 96% accuracy for binary classification of short-lived or long-lived patients. Pearson’s R (i.e., Pearson correlation coefficient) is 0.937 between survival time prediction and the actual clinical record. More importantly, the separate transcriptome encoder and drug encoder empower an in-silico optimal drug selection. Through a computational brute-force search, each patient is paired with every drug to test whether an alternative drug other than the actual prescribed one works better concerning survival time. In summary, our interpretable deep learning model enables precision medicine and has great potential to improve patient health.

## Results

### TCGA cancer genomics and clinical data

**Figure 1:**
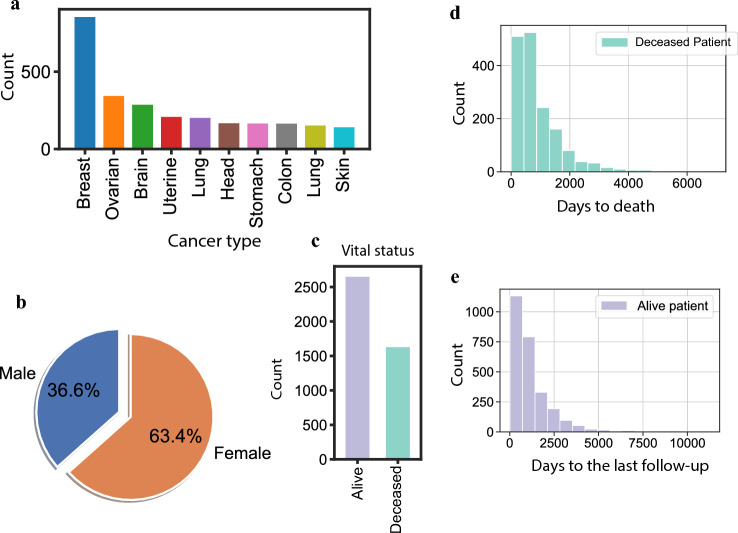
Overview of the TCGA data used in this study. (**a**) Top 10 cancer types from the TCGA cancer genomics data sorted by the number of patients. (**b**) Gender distribution. (**c**) Vital status from related clinical data. (**d**) Distribution of days-to-death for deceased patients. (**e**) Distribution of days-to-the-last-follow-up for alive and censored patients.

To predict the survival time of cancer patients based on their transcriptome profiles and drug treatments, we obtained the transcriptome and clinical data from TCGA. A total of 4311 patients have both transcriptome data and clinical information such as drug prescriptions and survival time, covering 178 drugs and 33 cancer types. The most abundant samples are from breast and ovarian tumors (Fig. 1a), and the gender distribution is 63.4% females and 36.6% males (Fig. 1b). Based on the vital status (Fig. 1c), 1645 patients (38.2%) were deceased. The distribution of their days-to-death is shown in Fig. 1d. For the other 2666 (61.8%) alive or censored patients, we used the days-to-the-last-follow-up (distribution shown in Fig. 1e) to infer whether a patient was long-lived. We trained a Gene Ontology (GO)-guided deep learning network to predict the impact of different drugs on the survival time given the patient’s gene expression profile. The actual number of patients used in our network training was slightly less, depending on whether the Morgan fingerprint of the drug is available.

### Two-stage training with knowledge transfer to fully utilize data

Transfer learning was initially devised to transfer the learner trained in similar but different domains to the target domain^20^. Recent studies show that two-stage training can improve deep learning models in the data-scarce setting^21,22^. Here the majority of available TCGA data are censored. Therefore, to harness the power of machine learning in personalized medicine, we devised a two-stage training schema in our CancerIDP with knowledge transfer.

In the first stage, the network weights are randomly initialized, and we aim to utilize as many patients as training samples as possible. To do that, we included both deceased and qualified censored patients to create binary classification supervision signals: long-lived or short-lived. Positive samples are long-lived patients known to be alive after 1,200 days, including patients with either days-to-death or days-to-the-last-follow-up greater than 1200. Negative samples are short-lived patients who died within 1000 days (i.e., days-to-death < 1000). The first stage's goal is to exploit as much data as possible to abstract deep representations from gene expression and drug structures for the vital status prediction (more straightforward binary classification), which will be used as a starting point in the second stage for fine-grind survival time prediction.

**Figure 2:**
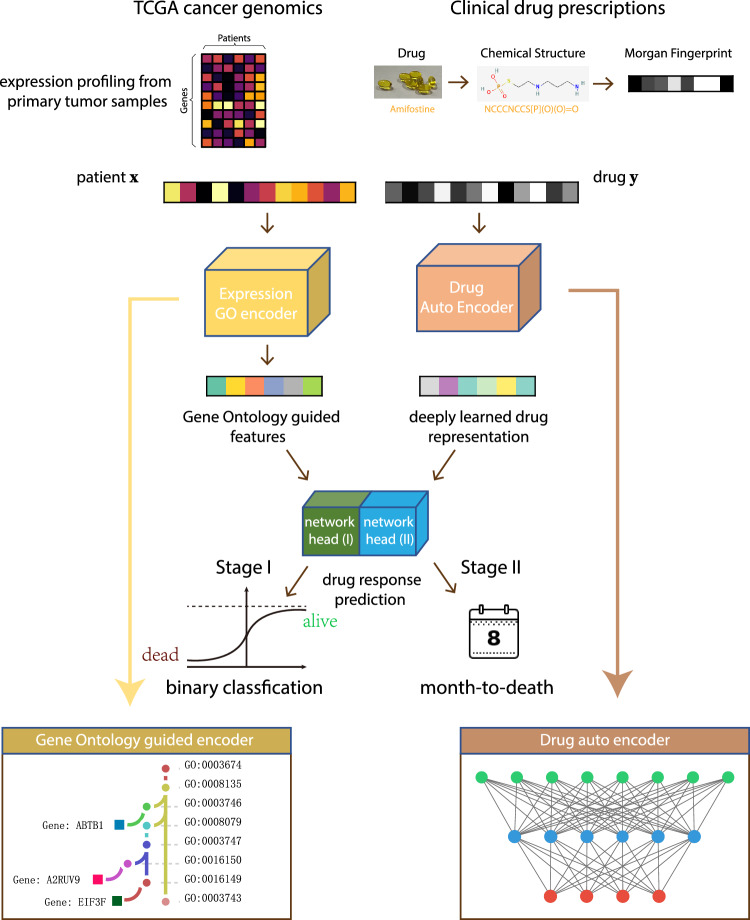
Interpretable network architecture design. Given a patient and the prescribed drug, the Gene Ontology-guided encoder takes the transcriptome as input to generate expression embedding. The drug encoder transforms the drug Morgan fingerprints into drug embedding. The network heads then concatenate the transcriptome and drug embeddings to predict the vital status in the first stage and the exact months-to-death in the second stage. The GO-guided expression encoder follows the same GO hierarchy in human cells.

**Figure 3:**
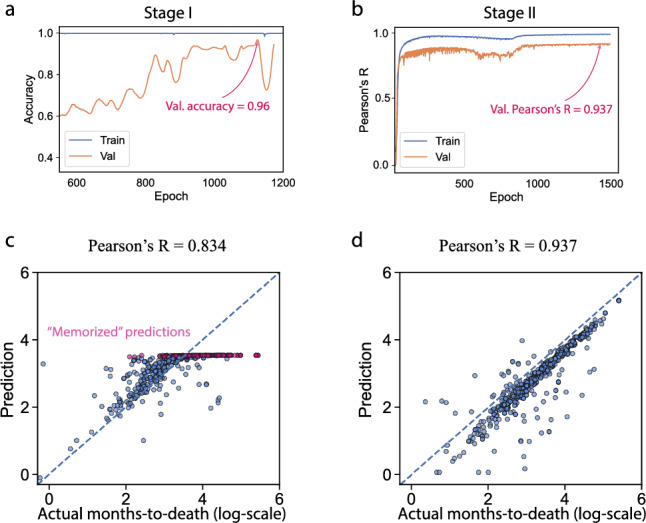
Performance evaluation and the memorization effect of the network. (**a**) The training and evaluation performance on hold-out validation data for vital status prediction (stage I). (**b**) The training and evaluation performance for survival time prediction (stage II). (**c**) The memorization effect for rarely prescribed drugs. Each dot represents a case. Red colored dots are those with rare drugs. (**d**) The performance improves after eradicating the memorized rare drugs. Pearson’s R increases to 0.937.

In the second stage, the network structure remains the same (Fig. 2), and the weights are initialized as those in the best-performing model on the validation set from the first stage. The assumption is that the feature extractor learned in the binary survival status classification can be a good starting point for a more fine-grind survival time prediction. Only deceased patients with exact days-to-death records are used in the second stage. The binary network head from the first stage is discarded, and a new regression head is trained from scratch to predict the days-to-death given a patient’s gene expression profile and the prescribed drug. In the actual training, the days-to-death was transformed to months-to-death on a log scale. Figure 3a shows that the best-performing model achieves 96% classification accuracy in the first stage on the hold-out data. In the second stage, Pearson’s R equals 0.937 between the ground truth months-to-death and the prediction (Fig. 3b).

### The memorization pitfall when predicting survival responses from drug treatments

Deep learning models have impressive expressiveness, but in practice, we noticed a potential pitfall that comes with the ability of the deep network to emulate any function. We called this the “memorization effect” in our survival prediction. In our earlier experiments, we included all drugs in the deep learning model and prepared the training and the hold-out validation sets by the random split. However, we observed that for some rarely prescribed drugs with only one or two supporting data points (i.e., 1–2 patients) in the training set, the network tends to simply “memorize” such drugs instead of generalizing meaningful embedding. And then, in the testing phase, the network just outputs the survival time of the patients that it has memorized (observed) in the training phase. Such effect is demonstrated in Fig. 3c: some of the predicted months-to-death values (log scale) are the same since the model only observed one patient with this specific drug during the training, and the model output the survival time of that patient in the testing when fed into this particular drug.

To counter the memorization effect, we eliminated drugs with less than five supporting data points in the training set. Although the number of training samples decreases slightly, the network does not suffer from the memorization effect but learns meaningful embeddings and is more generalizable. Pearson’s R improves from 0.834 to 0.937 after eradicating the memorization effect in the months-to-death prediction (Fig. 3c,d).

### Improving patients’ survival based on personalized medicine enabled by CancerIDP

Distinct from other deep-learning-based cancer survival time predictions, our model not only predicts survival time but also enables optimal drug selection for each patient based on the expression encoder and the drug encoder. From the existing clinical data, we know that patient *X* with transcriptome T is prescribed drug *D,* and the survival time is *S*. This triplet (*T*, *D*, *S*) forms a training data point for our network. With the predictive ability achieved through training, the network has learned to encode transcriptomes and drugs as predictive deep embeddings. We can then predict the drug response in silico by pairing each patient with each medication and identifying the optimal drug (D*) with the longest survival time for patient *X*.

**Figure 4:**
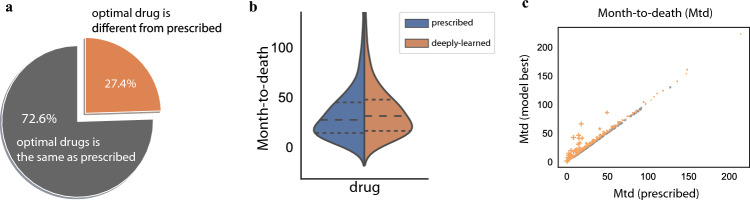
In-silico drug selection for cancer patients. (**a**) The percentages of patients with optimal drugs selected in-silico that are different from the actually prescribed drugs. (**b**) The distribution of the actual months-to-death compared with the predicted months-to-death under optimal drugs selected in-silico. The dotted lines mark the 25% and 75% quantiles, and the dashed lines denote the medians. (**c**) The months-to-death for optimal drugs compared with the actual months-to-death. Patients with a relative improvement of > 5% are colored in orange. The mark size is proportional to the relative improvement.

In this TCGA cancer genomics dataset, based on our in-silico drug selection, we can find a drug different from the actual prescription for 27.4% of patients, with which the predicted survival time is longer than the observed survival time (Fig. 4a). Figure 4b compares the actual survival time for the prescribed drugs and the predicted survival time if paired with the deeply-learned optimal drugs. Deep-learning-enabled personalized medicine can significantly improve patient survival by increasing the median of months-to-death from 27.1 to 31.0. Noticeably, 23.4% of patients can have a relative improvement in survival time by at least 5% (Fig. 4c, colored in orange, the size of the mark is proportional to the relative improvement). The most significant improvement is 2.97-fold, where the months-to-death is 24.7 for the prescribed drug and 73.4 for the optimal drug suggested in-silico.

To support our in-silico drug discovery, we randomly selected a patient (TCGA-ZH-A8Y4, patient 1 in Supplementary Fig. S1) and identified nine more patients with the most similar transcriptome profiling according to the cosine similarity between transcriptomes (normalized to unit vectors). Three of the ten patients had their predicted optimal drugs matching their actual prescribed drugs. Five drugs selected by CancerIDP were actually prescribed to at least one of the ten patients. Particularly, the drug Halichondrin B (canonical SMILE: CC1CC2CCC3C(=C)CC(O3)CCC45CC6C(O4)C7C(O6)C(O5)C8C(O7)CCC(O8)CC(=O)OC9C(C3C(CC4C(O3)CC3(O4)CC4C(O3)C(CC3(O4)CC(C4C(O3)CC(O4)C(CC(CO)O)O)C)C)OC9CC(C1=C)O2)C, PubChem CID: 44581725) was prescribed to patients 3 and 9, and predicted to be the optimal drug for patients 4, 5, and 9. After being prescribed Halichondrin B, patient 3 survived for 24.69 months, and patient 9 survived for 34.12 months. In contrast, patients 4 and 5 only survived for 18.21 and 29.45 months under different drug treatments. As predicted by CancerIDP, prescribing Halichondrin B could be beneficial for patients 4 and 5, as it may lead to longer survival times of 22.21 and 33.35 months, respectively, which is similar to its effect on patient 9. Thus, information obtained from CancerIDP could be used as input for an algorithmic expert system to aid clinical decision-making.

To further support our in-silico drug discovery, we also checked the newly approved cancer drugs since 2010 (summarized in^23^). These newly approved drugs usually increase cancer patients’ survival time. Our CancerIDP model included 46 patients involving these recently approved drugs. Of these, 35 patients had predicted drugs matching their actual prescribed newly approved drugs. Our analysis using CancerIDP showed that prescribing these newly approved drugs could increase the survival times of seven patients who were receiving other drugs. In contrast, CancerIDP predicted other drugs for only four patients who were prescribed these new drugs. These results may suggest that our CancerIDP predictions are more aligned with the recommendations of medical companies. Thus, deep representation learning provides great promise here. As the system is fed with more abundant data, such data-driven personalized drug prescriptions will become more valuable and trustworthy.

### Learn transcriptome embeddings that are predictive of months-to-death

The final prediction of the network is based on two components: the transcriptome embedding learned from the GO-guided encoder, and the drug embedding from the autoencoder. The drug autoencoder is simply a black-box model that learns representations from drug chemical structures expressed in the Morgan fingerprints. We are currently unable to interpret the mechanisms. However, our transcriptome embedding is based on the hierarchy structure of GO terms and has greater interpretability of how genes act together to predict drug survival responses.

**Figure 5:**
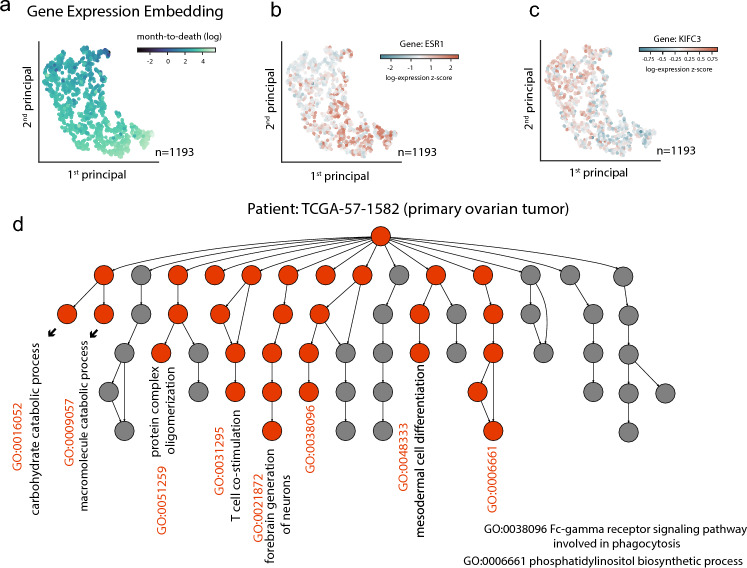
Visual inspection of gene expression embedding and the most discriminating GO entities. (**a**) Transcriptome embeddings learned in predicting months-to-death. The first two principal components from transcriptome t-SNE coordinates are shown. The color of the dots represents the months-to-death values. (**b**,**c**) Marker genes change along with the transcriptome embedding manifold: (**b**) gene ESR1; (**c**) gene KIFC3. The color of the dots represents expression levels. (**d**) The most discriminating GO terms based on attention weights. This illustrates an example from a patient with ovarian cancer.

We inspected the transcriptome embedding learned from all patients using t-SNE and visualized the top two principal components (Fig. 5a–c). The transcriptome embedding is informative about months-to-death, as shown in Fig. 5a. Patients with similar transcriptome embeddings share similar months-to-death values. The expression of some marker genes such as ESR1 (implicated in hormone resistance and anti-estrogen therapies for breast cancer, Fig. 5b) or KIFC3 (encodes a member of the kinesin-14 family of microtubule motors, Fig. 5c) is consistent with the spatial pattern of the transcriptome embedding, indicating the contribution of these genes to the transcriptome embedding for drug survival responses.

### Discover the most discriminating GO components for a cancer patient

Cellular responses of drugs depend on both transcriptomes and drug chemical structures^24^. In CancerIDP, we encode the expression information and drug information via transcriptome GO encoder and drug autoencoder (details in “Methods”). Since the neural network structure of the transcriptome GO encoder is designed according to the GO system structure in human cells, it is the key to the interpretability of CancerIDP. Specifically, we build the network based on the GO system. Each GO term (node) is represented by a multi-layer perceptron (MLP). These network nodes take inputs from child GO terms and send outputs to parent GO terms. The state of each GO term is represented by the activation of MLP, and the connectivity weights between nodes are determined through end-to-end network training. We applied the attention mechanism^25,26^ to learn the most important GO terms for survival time prediction (details in “Methods”). Attention weight assigned to each GO node indicates the relative importance of that GO term to the prediction task at hand. After training, when we forward pass a patient’s gene expression profile, we interpret the nodes with the highest attention weights as the most distinguished GO terms for survival determination for that patient.

Figure 5d shows an example of patient TCGA-57-1582 with ovarian cancer. The most discriminating GO terms are highlighted in red, including catabolic processes like carbohydrate catabolic process (GO:0016052), macromolecule catabolic process (GO:0009057), T-cell co-stimulation (GO:0031295), and some signaling pathways involved in phagocytosis. Therefore, such a GO-guided encoder informs the activated pathway at the individual level, paving the way for the mechanistic study of personalized treatment.

Similarly, we forward passed each patient’s gene expression to the trained GO encoder, and obtained attention weights of GO terms for each patient. We then summarized the most common GO terms with large average attention weights across patients. The discriminating GO terms with average attention weights across patients ≥ 0.15 are provided in Supplementary Table S1. Their positions in the pathway hierarchy are shown in Supplementary Fig. S2. Important GO terms include T cell mediated immune response to tumor cell (GO:0002424), negative regulation of G2/M transition of mitotic cell cycle (GO:0010972), extrinsic apoptotic signaling pathway via death domain receptors (GO:0008625), and so on.

## Discussion

Personalized medicine has great potential to significantly improve patient outcomes by taking into account an individual’s genomics information. Here, we developed an interpretable deep learning model CancerIDP on TCGA cancer genomics and clinical data. The data, covering patients diagnosed with 33 different tumor types and their treatment drugs, are publicly available from the National Cancer Institute’s (NCI) Genomic Data Commons (GDC) database. With proper training techniques, we proved that cancer survival time is highly predictable from high-dimensional transcriptomic data. A 96% accuracy can be achieved to distinguish between long- or short-lived patients. Pearson’s correlation coefficient on exact months-to-death between predictions and actual observations is 0.937. Our transcriptome encoder was designed to simulate the GO hierarchy in human cells. Instead of considering DNA point mutations from fixed cell lines like in DrugCell, our model takes gene expression data from individuals. Transcriptomes are dynamic and heterogenous, reflecting differences in genetics, environment, and lifestyle. They capture cellular changes caused by disease progression and therapy responses, while genetic data cannot. The application of transcriptomics in personalized medicine has rapidly emerged and made important impact to diseases such as cancers, cardiovascular diseases, and neurodevelopmental disorders^27^. Our incorporation of transcriptomes into the deep learning model further harnesses their power to yield transformative personalized medicine contributions.

While much progress has been made in improving the interpretability of DNNs, many of them focus on how different features of the input contribute to the output of the model^28,29^. Such techniques may be used to figure out which input genes are important for survival prediction. However, it is equally important or even more important to understand how these genes act together and contribute to patients’ survival time. The interpretability of our CancerIDP relies on the GO hierarchy in the model architecture design. This is totally different from attribution analysis or saliency analysis. The activating neurons point to the most discriminating GO pathways for each individual and inform us which molecular systems in a cell play a vital role in the patient’s survival prediction. When the system finishes training with high predicting capability, we presume the network forms an internal understanding of the relationship between patients’ survival, their transcriptomes, and drug chemical structures.

Based on the satisfactory prediction performance of CancerIDP, we hence propose an in-silico drug selection to search for a more desirable treatment. This in-silico drug optimization, combined with the interpretability of the GO structured expression encoder, provides data-driven precision medicine guidance for clinical practice. However, the implementation of personalized medicine is complex and multifaceted. More research involving collaborations between researchers and health providers is needed to fully realize its potential. Our work is the proof-of-concept of a drug selection algorithm powered by deep learning and computation. With more data becoming available in the future, more comprehensive frameworks can be developed. Even purely computational, the more advanced networks trained with more data will provide value in at least guiding drug design. It should be mentioned that in cases where a combination of drugs was used as a treatment, we treated each drug as an individual treatment. This limitation was due in part to the relatively small sample size of our training data. However, modeling the combination of drugs in-silico could be an interesting avenue for future research. Given that complex diseases such as cancer typically require combination therapies, this would be an important area to explore further.

In our cancer survival analysis, the survival time is the time from drug treatment to patient death. Real-life databases often record incomplete information and lack the exact survival time of some patients. When the survival time is unknown for a patient who lost clinical follow-up, such an observation is referred to as a censored data point. The last available time point is known as censoring time, usually days-to-the-last-follow-up. Censoring observations, however, contain useful information for cancer survival modeling as censoring time provides a lower bound for survival time. Such characteristic is common to real-life data: more data contain coarse information than precise information. This calls for advanced models to utilize the data fully.

Recently, two-stage (pre-train + fine-tuning) approaches^30,31^ were proposed to harness the deep learning models on data with such characteristics. In general, much data with coarse labeling are gathered to train simple classifiers in the pre-train stage. And then, knowledge obtained in the pre-train stage is transferred by sharing the feature extractor weights learned from the simple classifier. Finally, the learner is fine-tuned by training samples with fine-grind labeling. In the first stage of our model, we propose a binary classification such that patients known to be deceased within 1000 days of treatment are labeled short-lived (y = 0) and patients with a survival time lower bound (days-to-death or days-to-the-last-follow-up) greater than 1200 days are labeled as long-lived (y = 1). In doing so, some censored patients' cancer transcriptomics and clinical data can be incorporated into the network training. Thereby, we utilize the lower-bound information contained in the censored patients to its full potential. Then in the second stage, by sharing encoder weights, we transfer the learned knowledge for transcriptome and drug encoding and train a new network head for exact survival time prediction. In the second stage, only deceased patients with exact survival time labels are included. In our ablation study, we found that the Pearson's correlation coefficient for months-to-death prediction was 0.891 when trained with a single stage, and increased to 0.937 in the two-stage training paradigm.

Previous deep-learning models for survival analysis were applied for a single or small number of cancer types and did not consider drug and transcriptome data. These include colorectal cancer survival prediction based on histologic images and other clinical variables^15^, lung cancer survival prediction based on clinicopathologic variables^12^, and prediction for ten cancer types based on histopathologic images^32^. Our work considers all 33 primary cancer tumor types. This raises the question of whether the tissue type should be distinguished before training since tumor tissue type can be predictive to some extent for cancer survival^33^. We compared and found that when incorporating additional clinical information of the biopsies (sex, ethnicity, tissue of origin, and age at diagnosis) as additional features, the performance is on par with gene expression and drug embedding only. This indicates that the transcriptomic heterogeneity provides enough prognostic information for deep-learning-based survival prediction and that the embedding learned from high-dimensional expression data embraces human-interpretable knowledge, at least for common features such as genders and tissues. This makes our model innately simple: it requires no extra feature engineering or data processing efforts and favors end-to-end training. The only preprocessing we performed was a cross-sample normalization for the expression matrix. The end-to-end system also eliminates potential problems for clinical data since many patient data can be missing, and how to impute missing values in a deep-learning system post significant challenges for network training^34,35^.

In summary, our CancerIDP demonstrates the great potential of deep learning models to improve cancer patient survival based on personal transcriptomes in precision medicine. Furthermore, with unprecedented amounts of data available in the near future, deep learning models will further enhance the predictive performance and derive essential values from the data to benefit health through such personalized approaches.

## Methods

### Clinical drug data

Both clinical data and transcriptome profiling RNA-seq data used in this paper for TCGA were obtained from the GDC data portal (https://portal.gdc.cancer.gov/repository). Clinical supplement files for the human cancer dataset in BCR biotab format were downloaded from the GDC portal. Drug prescription, if available, for each TCGA patient was logged in those clinical supplements. Raw data cover 12,571 patients and 384 drugs. We first removed patients without clinical information and drugs without matched standard names, resulting in 4431 patients and 202 unique drugs. We standardized drugs by querying the GDISC database^36^, which was established by querying PubChem entries for each compound. Among the filtered drugs, 178 of them have at least five survival data points. For patients (103 such patients) that had a tandem usage (different drugs prescribed chronologically) of multiple drugs or prescribed with a combination of drugs at the same time, we pair the patient’s transcriptome profile with each drug signature to be used as a training data point. To further convert drug data to a quantitative representation that can be used in the network encoder, each drug is firstly converted to a simplified molecular-input line-entry system (SMILES) representation of its chemical structure via PubChem (https://pubchem.ncbi.nlm.nih.gov) and then encoded as a numerical vector with a fixed length (n = 2048) of the Morgan fingerprint. The conversion of the SMILES representation to the Morgan fingerprint of drugs was performed with the open-source Python library RDKit.

### Expression data

Transcriptome profiling via RNA-seq was used as expression data. We used Fragments Per Kilobase of transcript per Million mapped reads (FPKM) expression measurements for samples with “Sample Type” being “Primary tumor.” We focus on the 3008 genes identified from the top 15% most frequently mutated genes in Cancer Cell Line Encyclopedia (CCLE)^37^ that were annotated to Gene Ontology (GO)^38^ terms. This list of genes is also referred to as “DrugCell genes”^8^. Input normalization is extensively used in deep learning models to facilitate gradient learning and speed up convergence. Compared to raw FPKM values, standardized FPKM values across individuals (subtracting its mean and dividing by its standard deviation) speed up training and convergence and improve the final performance by 1%. The network we described in the main text was trained with the standardized FPKM.

### Survival data

Based on the vital status of the patients, we designed two experiments. The first was designed as a binary classification, with patients known to be deceased within 1000 days as short-lived (y = 0) and patients known to be alive after 1200 days as long-lived (y = 1). A total of 2399 patients were included (1121 short-lived patients and 1278 long-lived patients). The second experiment is a more fine-grind model, wherein we used all deceased patients (a total of 1631 patients) to train the model to predict the clinical months-to-death data recorded for each patient.

### In silico optimal drug selection

To perform the in silico optimal drug selection for every patient, we fed in each of the drugs to our CancerIDP. Thus, no matter whether the patient had ever taken the drug or not, we paired the drug’s chemical structure and the patient’s transcriptome expression and fed into the model. The predicted survival time was recorded. The drug corresponding to the longest predicted survival time was selected as the optimal drug for the considered patient.

### Neural network CancerIDP construction

The neural network is composed of two branches. The transcriptome GO encoder takes the log-transformed expression values as input and produces expression embedding. The drug autoencoder takes the Morgan fingerprints of drugs and learns the deep representation of drugs. The transcriptome GO encoder is built by mimicking the directed acyclic graph (DAG) formed by GO terms with the 3008 cancer genes as leaf nodes. The GO term architecture is based on DrugCell^8^. From the leaf nodes (gene expression values) to the root formed by the GO hierarchy, each internal node (GO term) is modeled with k (k = 6) neurons through a linear layer and a batch normalization layer^39^. And the input to the linear layer is the concatenation of all child states of the internal nodes. The drug autoencoder has no prior information injected and is realized by a multilayer perceptron with three layers and the number of neurons for each layer is 64, 32, and 4. Finally, the network head combines the gene expression embedding and deeply-learned drug representation to predict the binary long- or short-lived status or the months-to-death via a non-linear classifier.

### Identification of the most discriminating GO terms

The gene expression encoder is structured according to the GO hierarchy in human cells. The internal state of each term is finally brought down to a single neuron value with a neuron activation ReLU^40^. A standard attention mechanism was applied to the child nodes (genes or child terms in the GO hierarchy) when computing the hidden representation for parent nodes (leaf or internal GO terms). This approach helps us track and interpret the important child nodes in relation to calculating the parent value. Specifically, for the root and depth-1 terms (where each node has many children), we consider the child nodes with an attention weight > 0.05 as the most discriminating GO terms. For depth-2 terms, we consider an attention weight > 0.1 as most discriminating, and for depth-3 to leaf terms, an attention weight > 0.2 is considered most discriminating.

## Supplementary Information

Supplementary Information.

## Data Availability

All data analyzed in this study are described in the article. The source codes and detailed analysis steps are available at https://github.com/bsun0802/CancerIDP.
