# Supplementary material for: Interpretable deep learning for improving cancer patient survival based on personal transcriptomes
Source: Sci Rep. 2023 Jul 13;13:11344. doi: 10.1038/s41598-023-38429-7 (PMC10344908; doi:10.1038/s41598-023-38429-7)

Supplementary Table S1: Discriminating GO terms with average attention weights across patients ≥ 0.15 in CancerIDP.

id	level	depth	name	average_attn_weight
GO:0002424	level-04	depth-06	T cell mediated immune response to tumor cell	0.366562
GO:0010972	level-08	depth-09	negative regulation of G2/M transition of mitotic cell cycle	0.253937
GO:0008625	level-05	depth-07	extrinsic apoptotic signaling pathway via death domain receptors	0.253122
GO:0002026	level-03	depth-07	regulation of the force of heart contraction	0.246387
GO:0010882	level-06	depth-09	regulation of cardiac muscle contraction by calcium ion signaling	0.228325
GO:0002026	level-03	depth-07	regulation of the force of heart contraction	0.218206
GO:0051482	level-04	depth-04	positive regulation of cytosolic calcium ion concentration involved in phospholipase C-activating G protein-coupled signaling pathway	0.216494
GO:0034162	level-06	depth-013	toll-like receptor 9 signaling pathway	0.215577
GO:0035162	level-05	depth-05	embryonic hemopoiesis	0.214366
GO:0051259	level-06	depth-06	protein complex oligomerization	0.197241
GO:0002026	level-03	depth-07	regulation of the force of heart contraction	0.192962
GO:0034162	level-06	depth-013	toll-like receptor 9 signaling pathway	0.189351
GO:0030163	level-04	depth-05	protein catabolic process	0.180288
GO:0031295	level-03	depth-09	T cell costimulation	0.179447
GO:0016052	level-04	depth-04	carbohydrate catabolic process	0.176411
GO:0043543	level-05	depth-06	protein acylation	0.175249
GO:0030163	level-04	depth-05	protein catabolic process	0.174804
GO:0061061	level-03	depth-03	muscle structure development	0.170272
GO:0048333	level-04	depth-04	mesodermal cell differentiation	0.169472
GO:0006355	level-06	depth-09	regulation of DNA-templated transcription	0.169211
GO:0050796	level-06	depth-09	regulation of insulin secretion	0.168778
GO:0030163	level-04	depth-05	protein catabolic process	0.168761
GO:0044248	level-03	depth-03	cellular catabolic process	0.168129
GO:0010613	level-06	depth-08	positive regulation of cardiac muscle hypertrophy	0.167529
GO:0042059	level-07	depth-08	negative regulation of epidermal growth factor receptor signaling pathway	0.167182
GO:0060627	level-04	depth-05	regulation of vesicle-mediated transport	0.16698
GO:0031175	level-06	depth-06	neuron projection development	0.166801
GO:0050900	level-02	depth-04	leukocyte migration	0.166562
GO:0007585	level-02	depth-02	respiratory gaseous exchange by respiratory system	0.16626
GO:0098609	level-03	depth-03	cell-cell adhesion	0.165595
GO:0050996	level-06	depth-07	positive regulation of lipid catabolic process	0.164903
GO:0007229	level-04	depth-06	integrin-mediated signaling pathway	0.164763
GO:0007189	level-05	depth-07	adenylate cyclase-activating G protein-coupled receptor signaling pathway	0.16251

GO:0090130	level-02	depth-02	tissue migration	0.16085
GO:0019722	level-05	depth-07	calcium-mediated signaling	0.160417
GO:0002067	level-06	depth-06	glandular epithelial cell differentiation	0.158361
GO:0001819	level-05	depth-07	positive regulation of cytokine production	0.158175
GO:0038083	level-07	depth-09	peptidyl-tyrosine autophosphorylation	0.155831
GO:0006656	level-05	depth-08	phosphatidylcholine biosynthetic process	0.155294
GO:0034142	level-08	depth-013	toll-like receptor 4 signaling pathway	0.15213
GO:0038128	level-07	depth-09	ERBB2 signaling pathway	0.150415
GO:0060999	level-05	depth-05	positive regulation of dendritic spine development	0.149949
GO:0007616	level-05	depth-07	long-term memory	0.149661

Supplementary Figure S1. Months-to-death prediction for 10 patients with similar transcriptomes. We randomly selected a patient (TCGA-ZH-A8Y4, top row) and identified nine more patients with the most similar transcriptome profiling according to the cosine similarity between transcriptomes (normalized to unit vectors). The black box indicates the best in-silico predicted drug, and the green box shows the actual prescribed drug for a patient. Spearman correlation of rows 2-9 to row 1 ranged from 0.67 to 0.84. In contrast, the drug prediction had a correlation of only 0.31 with another randomly selected patient.

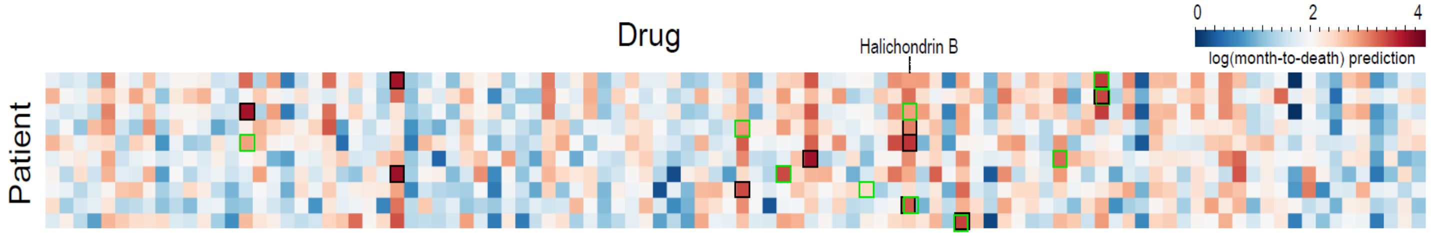

Supplementary Figure S2. Positions of recurrent discriminating GO terms in the pathway hierarchy. A radial layout of the GO-guided gene expression encoder is shown. Each node represents a GO term. Only the edges of a depth-first search tree of the complete GO structure are shown for visual convenience. The node color represents the average attention weight across patients.

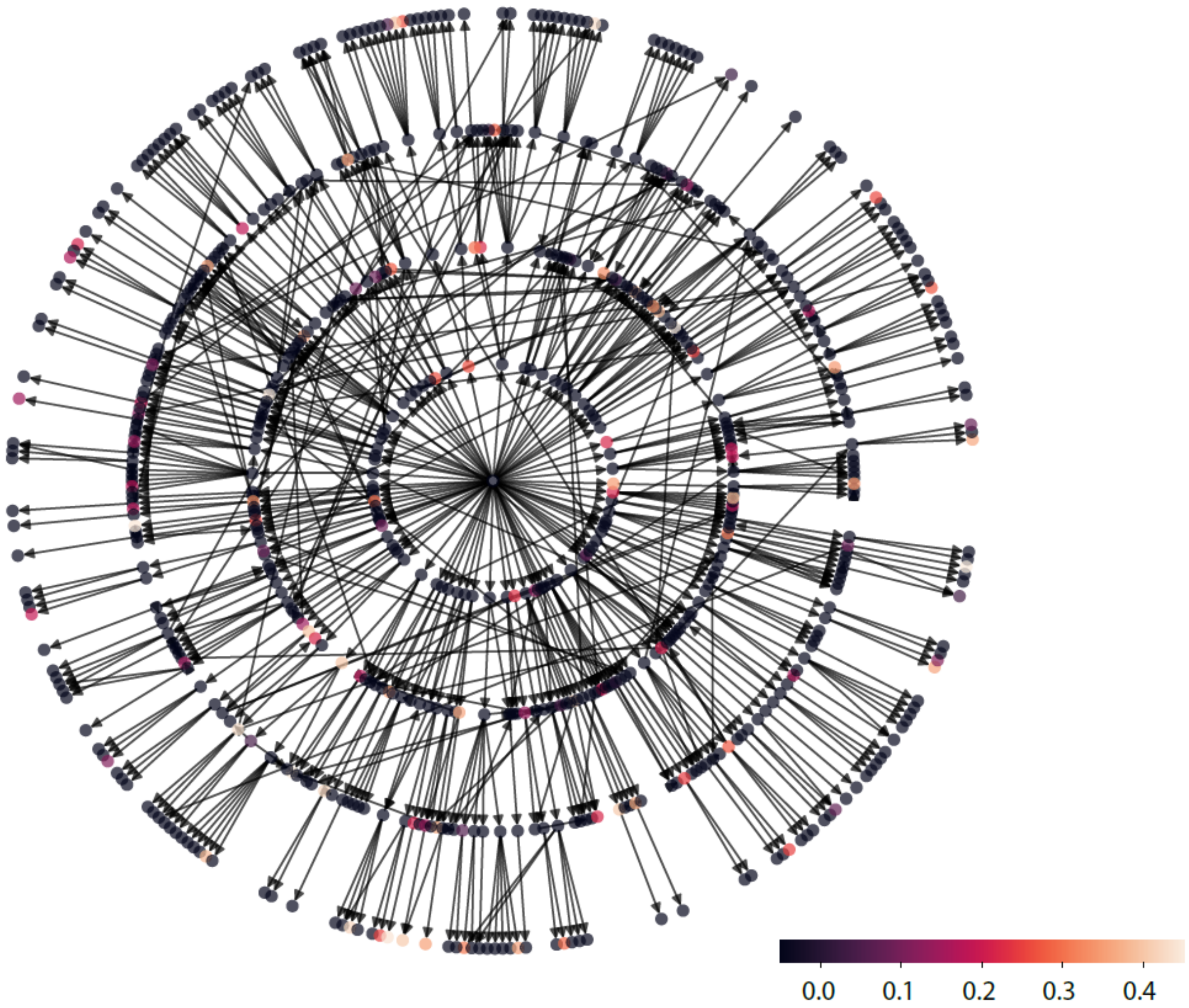

Supplement: Supplementary file 1 — Supplementary Information. [file 41598_2023_38429_MOESM1_ESM.pdf]
